# A Protein Concentration Measurement System Using a Flexural Plate-Wave Frequency-Shift Readout Technique

**DOI:** 10.3390/s130100086

**Published:** 2012-12-21

**Authors:** Chua-Chin Wang, Tzu-Chiao Sung, Chia-Hao Hsu, Yue-Da Tsai, Yun-Chi Chen, Ming-Chih Lee, I-Yu Huang

**Affiliations:** 1 Department of Electrical Engineering, National Sun Yat-Sen University, Kaohsiung 80424, Taiwan; E-Mails: ccwang@ee.nsysu.edu.tw (C.-C.W.); song@vlsi.ee.nsysu.edu.tw (T.-C.S.); ydtsai@vlsi.ee.nsysu.edu.tw (Y.-D.T.); oieuc@vlsi.ee.nsysu.edu.tw (Y.-C.C.); d943010029@student.nsysu.edu.tw (M.-C.L.); 2 Industrial Technology Research Institute (ITRI), Tainan 70955, Taiwan; E-Mail: ChiaHaoHsu@itri.org.tw

**Keywords:** FPW, frequency-shift readout circuit, peak detection, resonant frequency, immunoglobulin E

## Abstract

A protein concentration measurement system with two-port flexural plate-wave (FPW) biosensors using a frequency-shift readout technique is presented in this paper. The proposed frequency-shift readout method employs a peak detecting scheme to measure the amount of resonant frequency shift. The proposed system is composed of a linear frequency generator, a pair of peak detectors, two registers, and a subtractor. The frequency sweep range of the linear frequency generator is limited to 2 MHz to 10 MHz according to the characteristics of the FPW biosensors. The proposed frequency-shift readout circuit is carried out on silicon using a standard 0.18 μm CMOS technology. The sensitivity of the peak detectors is measured to be 10 mV. The power consumption of the proposed protein concentration measurement system is 48 mW given a 0.1 MHz system clock.

## 1. Introduction

With the booming demand in the field of biomedical electronics, *in vitro* bio-analytical applications are being quickly developed to help medical staff perform point-of-care (POC) pathologic analysis [1–3]. Protein concentration measurements are important analysis methods, widely adopted to diagnose the presence of illnesses. For instance, many people suffer from different allergic diseases, e.g., allergic rhinitis, which may cause extremely uncomfortable feelings and poor quality of life. Clinically, the concentration of immunoglobulin E (IgE) protein is one of the important indicators to reveal the allergic level in human serum [4–8]. Many conventional commercial allergy measurement techniques are available to analyze IgE concentration, e.g., enzyme-linked immunosorbent assay (ELISA) [9], surface plasmon resonance (SPR) [10], quartz crystal microbalance (QCM) [11] sensing techniques, chemiluminescence immunoassay (CLIA) [12], and Abbott AxSYM fluorescence polarization immunoassay (FPIA) [13], *etc.* Unfortunately, all the commercial allergy measurement instruments mentioned above require multifarious testing protocols, long operation time for sampling analysis procedures, expensive analytical instruments, and many analysts. These equipments might not be available in remote areas, or even those clinics in suburban areas. Therefore, a new inexpensive allergy detector with short analysis time and high precision is very much needed for those who suffer these areas.

## 2. Design and Fabrication of the Proposed FPW Biosensor

### 2.1. Biosensor Microfabrication Technology

**Figure 1.**
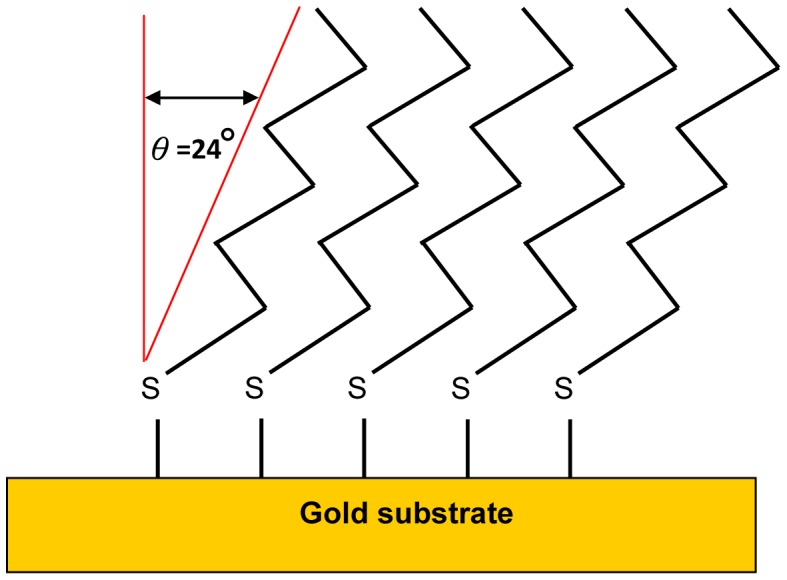
Proposed structure for the SAM.

Molecular self-assembly monolayers (SAMs) are spontaneous organizations of molecules into stable, structurally well-defined aggregates. The basic principles of molecular self-assembly are found throughout biology: protein folding and aggregation and pairing of base pairs in DNA are two well-known examples. SAMs were preceded historically by Langmuir-Blodgett (LB) monolayers, which have been studied extensively and are useful for many applications. LB films, however, are neither convenient to prepare nor sufficiently robust for most applications. SAMs, by contrast, are more robust and simpler to generate, inamuch that they can be formed from a wide variety of ligands and supports. SAMs can be immobilization on Pt [14,15], Cu [16–19], Ag [10,20], and Au [11,12,21] metal surfaces. In this study, we adopt Au as an experimental biomolecule substrate based on the interaction between dilute solutions of thiols (or disulfides) and gold surfaces to form Au-S bonds. The sulfur in the thiol (S–H) group has a strong chemical interaction and orients on the gold surface owing to the accommodation of the optimal van der Waals interactions between adjacent molecules. Hence, these chains forms with a fixed tilt. As a result, *n*-hexadecane thiol chains orient themselves into aligned monolayers 20–25° above the Au (111) surface, and the chains adjust their spacing to optimize the van der Waals interaction [20], as shown in Figure 1.

**Figure 2.**
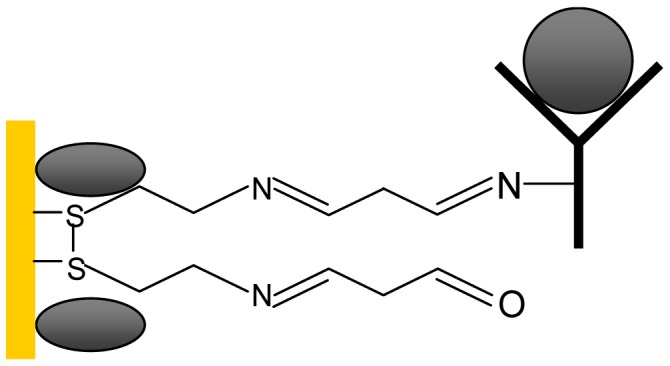
Schematic diagram of the integration of cystamine SAM.

IgE antibodies exploit SAMs to immobilize on the flexural plate-wave (FPW) sensor surface, which is required to achieve high selectivity of an immunosensor. Utilizing the highly specific IgE antigen-antibody interaction is a good candidate for the IgE molecular recognition process. To ensure IgE molecules with low non-specific binding of other biomolecules, an appropriate self-assembled monolayer must be developed. In this study, the well-bonded cystamine SAM/glutaraldehyde cross-linking layers are proposed for the immobilization of the IgE antibody. The highly ordered cystamine SAM can be nucleated on a (111) gold surface and utilized for modification of a gold surface to introduce amino groups on it. The amino groups can provide reaction sites for covalently bonding with glutaraldehyde. The purified mouse anti-human IgE antibodies are then immobilized through Schiff bases via glutaraldehyde cross-linking, as shown in Figure 2. Furthermore, this work utilizes MEMS and IC technologies to develop an IgE biosensing microsystem which is a total solution with small test sample volume, short operation time, small physical size and low cost.

In the last decade, many acoustic microsensors have been developed for molecular mass detection, such as shear horizontal surface acoustic wave (SH-SAW), surface transverse wave (STW), love wave (LW), shear horizontal acoustic plate mode (SH-APM), layered guided acoustic plate mode (LG-APM) and FPW [22–27]. However, except for the FPW device, the operating frequency of the above mentioned acoustic microsensors is usually larger than 90–100 MHz, which results in difficulties of readout IC design and development of portable microsystems [26,27]. Furthermore, since the phase velocity of the FPW device is less than the sound velocity in liquid, only a small portion of the energy will be dissipated into the testing liquid. Even though the fabrication cost of conventional SAW devices is much lower than that of the FPW devices, the low frequency (<10 MHz) and high mass sensitivity (>8 × 10^7^ cm^2^·g^−1^) of FPW devices makes them more appropriate for applications in clinical, industrial, environmental and biological detection using liquid samples than other acoustic microsensors [26,27]. Three characteristics of the FPW devices make them a very good choice for biomedical analysis, which are listed as follows [21,22]:

1. Low energy loss of acoustic transmission in liquid medium
2. Low design complexity of the oscillator
3. Isolating the electrical circuit from the liquid medium

Besides, since the FPW device can be fabricated by semiconductor technology, which allows batch processing that is very cost effective. Generally, the structure of the FPW device is composed of a group of input interdigital transducers (IDTs), a group of output IDTs, and an acoustic waveguide material between the input IDTs and the output IDTs. Notably, the acoustic waveguide material is made of a piezoelectric material. The FPW device's performance is determined by the quality and the membrane thickness of the piezoelectric layers. Therefore, the FPW device needs an excellent micro-fabrication technique to be realized by the microelectromechanical systems (MEMS) and piezoelectric films. The mechanical deformation of the piezoelectric material is caused by the piezoelectric effect when the signal is given by the input IDTs, which generates an acoustic wave at the input IDTs in the meantime. Then, the acoustic wave is propagated to the output IDTs through the piezoelectric films and restored into an electrical signal by the piezoelectric effect. To improve the performance of the FPW device, piezoelectric films with uniform microstructures and textures are a basic requirement. Moreover, the smoothness of the piezoelectric films would also enhance the acoustic transmission efficiency. Table 1 shows the comparison of the commercial allergy measuring methods and the FPW-based microsystem. Obviously, the FPW-based microsystem offers the advantages of low cost and short operation time. Moreover, another advantage is that the FPW-based microsystem requires very few samples of human serum.

### 2.2. FPW Allergy Biosensor Structure and Characteristics

**Figure 3.**
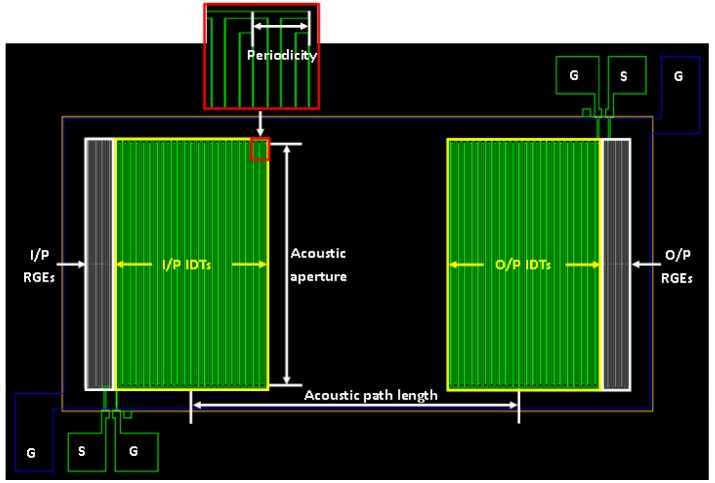
Layout of the FPW biosensor with grating electrode design.

The FPW allergy biosensor propagates an acoustic wave via a mechanical thin plate. The resonant frequency, *f_0_*, of the FPW sensor is given by the following equation:

(1) $$f_{0}=\frac{V_{P}}{\lambda }$$

where *λ* is the acoustic wavelength and *Vp* is the phase velocity [27]. The mass loading of the Si/SiO_2_/Si_3_N_4_/Cr/Au/ZnO floating thin plate which results in resonant frequency shift is expressed as follows:

(2) $$\frac{\Delta f}{f_{0}}=S_{m}\Delta m=S_{m}(MW\times C_{s})$$

where *Δf* is the change of the resonant frequency due to a change in mass per unit area, *Δm*, and *S_m_* is the mass sensitivity of the FPW allergy biosensor. The design specifications of the IDTs of the FPW sensor are listed in Table 2 and the corresponding layout diagram is illustrated in Figure 3. The grating electrode adopted in this research is designed to effectively reduce the insertion loss of FPW sensor.

**Figure 4.**
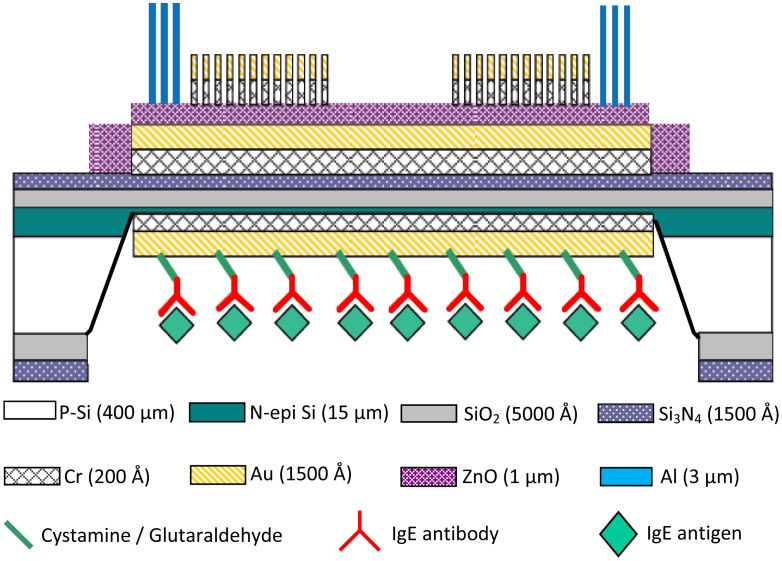
Cross-sectional structure of the FPW-based allergy biosensor.

Figure 4 schematically shows the cross-sectional structure design of the FPW-based allergy biosensor, where the cystamine SAM/glutaraldehyde/IgE antibody/IgE antigen multilayer is integrated on the backside Au electrode.

**Figure 5.**
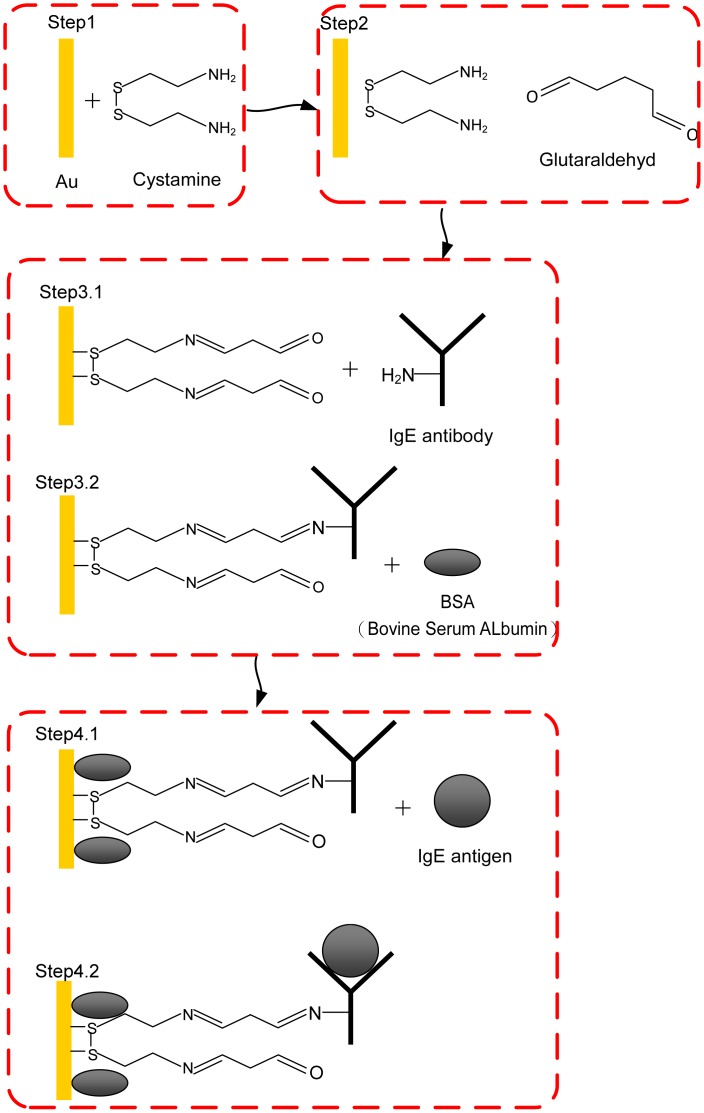
Schematic diagram of the integration of cystamine SAM, glutaraldehyde and IgE antibody/antigen in multilayers.

To improve the adhesion between the gold layer and the silicon substrate, a 500 Å-thick Cr layer is deposited onto the backside cavity of the FPW device before the 2,500 Å-thick Au layer deposited by an E-beam evaporator. To achieve a hydrophilic surface, the backside gold layer is pretreated with ‘piranha’ solution (70 wt% H_2_SO_4_–30 wt% H_2_O_2_) for 30 min, and then rinsed with deionized (DI) water three times and dried at the room temperature. An immunoaffinity layer for the effective recognition of IgE antigens is generated through the following steps [26] and a graphical illustration is given in Figure 5.

*Step 1*: The chip is treated with a cystamine solution (0.02 M) for 1 h, washed with DI water three times and air-dried.
*Step 2*: The chip is dipped into 2.5 wt% aqueous glutaraldehyde cross-linking reagent for 1 h and washed with DI water three times and air-dried.
*Step 3*: After the highly purified, the IgE antibody layer is coated on the surface of the backside glutaraldehyde layer, and injecting Tween-20 wash buffer three times.
*Step 4*: A diluted bovine serum albumin (BSA) layer is used for blocking and incubating the IgE antibody-coated surface to avoid nonspecific absorption and injecting diluted human IgE antigen.

By the above steps, the purified mouse anti-human IgE antibodies are then immobilized through Schiff base via glutaraldehyde cross-linking.

**Figure 6.**
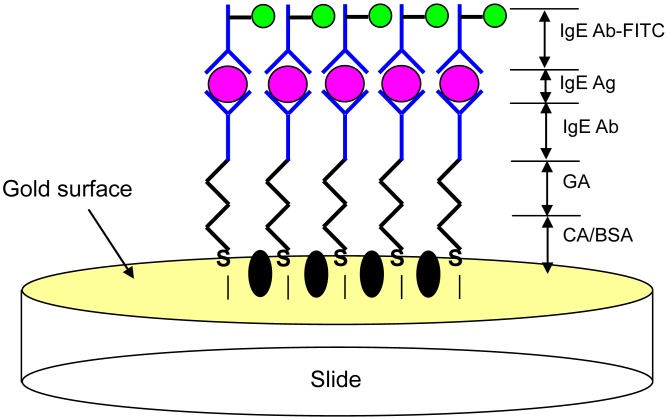
Schematic diagram of the integration of cystamine, glutaraldehyde, IgE antibody/antigen, and IgE antibody-FITC on the slide.

**Figure 7.**
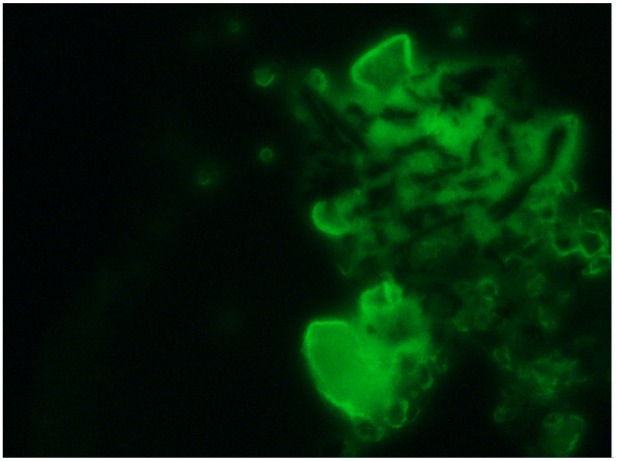
Fluorescence microscopic image of sandwich format (with IgE antigen concentration 294 IU·mL^−1^) on the gold/glass microscope slide.

In this section, we investigate the sandwich format shown in Figure 6, which is produced using a fluorescence microscope measurement to generate real-color photographs. The detailed procedures related to the immobilization process are described in the steps above. In order to investigate the fluorescence microscope images of sandwich format, we add the following steps: goat anti-human IgE-fluorescein isothiocyanate (FITC) are attached to the antigen/antibody pairs on the surface of a gold/glass microscope slide via a glutaraldehyde layer with a reaction time of 1 h, which may be seen as transparent. The above procedures were performed at room temperature in a dark environment. This operation of the fluorescence microscope is described as follows: laser beams with a wavelength of 488 nm increase the electronic transition energy of FITC fluorescein molecules, enabling electronic resonance to occur within the molecules; hence, these FITC molecules emit at an optical wavelength of 520 nm, which passes through an emission filter. The images were recorded using a digital camera [26]. According to the size and brightness of the green dots in the image, the practical IgE antigen concentration can be estimated. The green dots in Figure 7 show an IgE antigen concentration of 294 IU·mL^−1^.

### 2.3. FPW Allergy Biosensor with Frequency-Shift Readout IC Microsystem

In this paper, we propose a novel frequency-shift readout system for a pair of two-port FPW biosensors. The FPW allergy biosensor adopts the Cr/Au-based IDTs to be a transmitter (Tx) and a receiver (Rx), which are, respectively, placed on the right and left side of a thin plate. The sensitivity of the FPW allergy biosensor is −4.68 × 10^9^ cm^2^/g according to previous results [27]. Notably, the resonant frequency shift of the FPW allergy biosensor is roughly inversely proportional to the purified human IgE antigen concentration. Therefore, the FPW-based allergy biosensor provides another IgE antigen concentration measurement method. The proposed frequency-shift readout system is realized by a standard 0.18 μm CMOS technology. When the input frequency equals to the resonant frequency, the output signal amplitude of the FPW allergy biosensor will be maximum according to the resonant principle. Therefore, a high sensitive peak detector is required to detect the maximum peak voltage. Then, the peak detector generates an enable signal to trigger a register to snapshot the frequency value from the oscillator control signal. Thus, the frequency-shift value is attained. The amount of the IgE antigen concentration can be calculated according to the measured frequency shift between resonant frequencies of sensor1 (Experimental group, with antigen) and sensor2 (Control group, without antigen). The power consumption of the proposed frequency-shift readout circuit is measured to be 48 mW given by a 0.1 MHz system clock.

## 3. Frequency-Shift Readout Circuit

### Frequency Shift Readout Circuit Design

**Figure 8.**
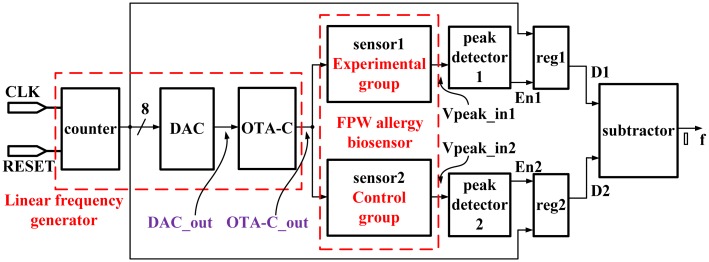
Block diagram of the frequency-shift readout system.

As mentioned, a frequency-shift readout circuit is needed to measure the amount of the frequency shift according to the characteristics of the FPW allergy biosensor. Figure 8 shows the proposed frequency-shift readout system, which is composed of an 8-bit up-counter, an 8-bit digital-to-analog converter (DAC), an operational transconductance amplifier-converter (OTA-C) oscillator, a pair of peak detectors, two registers, and a subtractor. The detailed description of each subcircuit is explained in the following text.

#### Linear Frequency Generator

**Figure 9.**
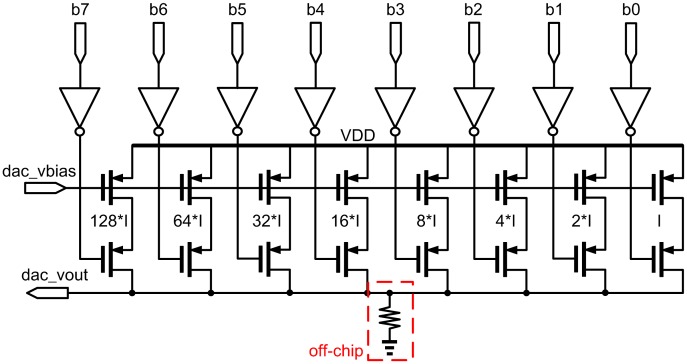
Schematic of the 8-bit DAC.

A sine wave frequency generator is required for the FPW allergy biosensors to generate the frequency sweep signal in the pre-defined range. Referring to Figure 8, the counter is a typical digital 8-bit counter generating a counting signal from 0 to 256 to drive the DAC. The 8-bit DAC utilizes a current-steering structure as shown in Figure 9, which requires only eight current sources with binary-weighted sizes instead of 2^8^ − 1 sources. Notably, the advantage of the binary weighting method is that the decoder is no longer needed by using the counter output as the input signal directly. In other words, it is easy to control and consume less area on silicon.

**Figure 10.**
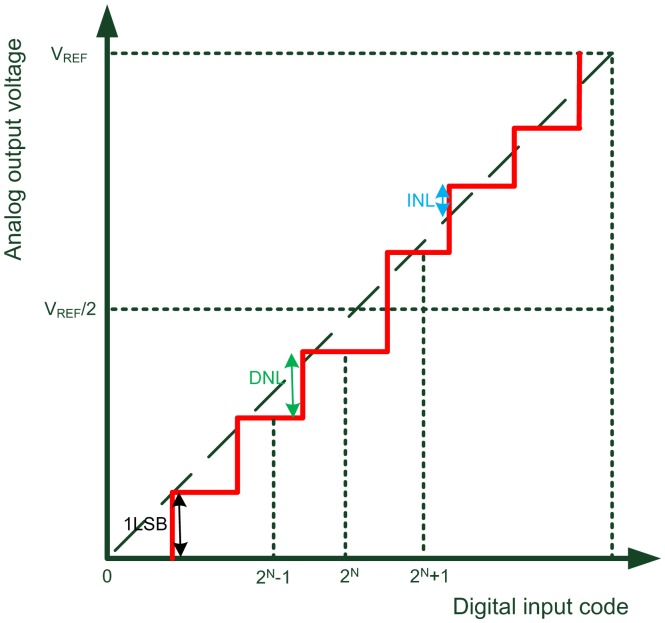
Illustration of DNL and INL error (Adapted from [29]).

**Figure 11.**
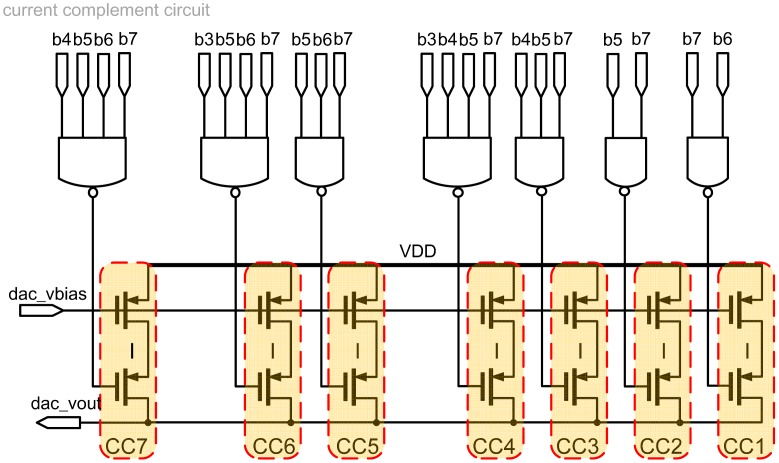
Schematic of the current complementary circuit.

**Figure 12.**
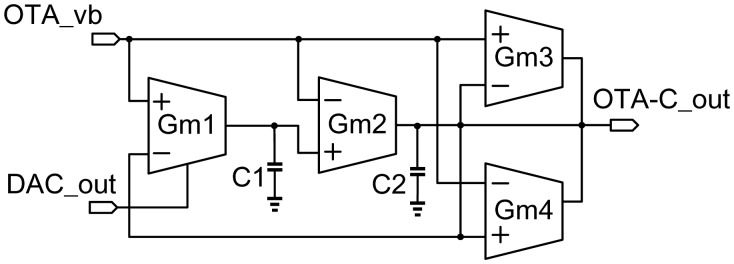
Schematic of the OTA-C oscillator.

However, the codes to drive the DAC, particularly the most significant bits (MSB), are easily affected by the process variation [28] to cause the current source mismatch. When the DAC output voltage rises gradually, some of the transistors will no longer operate in the saturation region such that the total output current is smaller than what is expected, which in turn causes serious integral non-linearity (INL) and differential non-linearity (DNL). A graphical illustration of DNL and INL is shown in Figure 10. To reduce the process variation causing the nonlinearity of the 8-bit DAC output voltage, a current complementary circuit (CC) is proposed to generate a corresponding complementary current to the DAC output, as shown in Figure 11. The current complementary circuit compensates the current directly by biasing the voltage of the MSB current paths [29]. That is the magnitude of the complementary current is tuned by DAC_bias, which is driven by a typical bandgap bias circuit (not shown) and adjusted by an off-chip resistor to ensure that INL and DNL will be less than 0.5 LSB [29]. Therefore, given an input binary code, DAC out can be set to an appropriate voltage level for the following OTA-C oscillator. The schematic of the tunable OTA-C oscillator is shown in Figure 12 [30].

**Figure 13.**
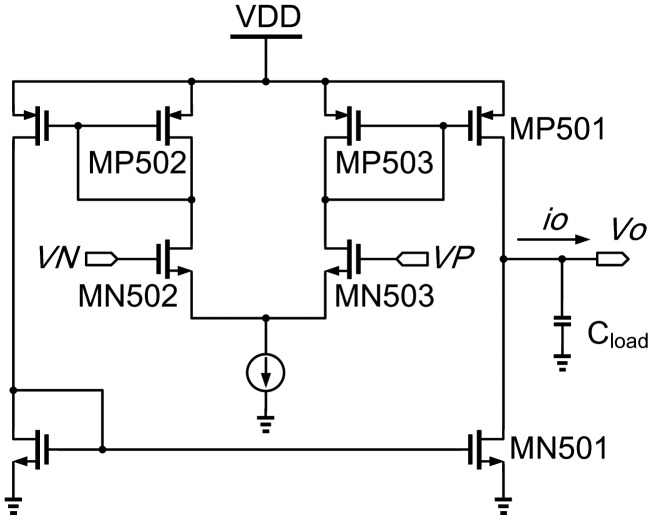
Schematic of the operational transconductance amplifier.

OTA_vb is biased externally to ensure correctness of the OTA's functionality. Gm1, Gm2, Gm3, and Gm4 are identical OTA (operational transconductance amplifier) like the one shown in Figure 13. Assuming MP502 = MP503 and MN502 = MN503, the drain-source currents of MP502 and MP503, *i_d,P502_* and *i_d,P503_*, are expressed as follows:

(3) $$-i_{d,P502}=i_{d,P503}=\frac{g_{m}}{2}(\text{VP}-\text{VN})$$

where *g_m_* is the transconductance of MP503, respectively. Furthermore, the gain of the OTA is governed by the following equation:

(4) $$A_{V}=\frac{V_{O}}{\text{VP}-\text{VN}}=g_{m}(r_{o,P501}\backslash \backslash r_{o,N501})$$

where *r_o,P501_* and *r_o,N501_* are output impedance of MP501 and MN501, respectively. If the impedance of the output load is smaller than OTA output resistance at a high frequency, the output current, *i_o_*, can be written as follows:

(5) $$i_{o}=i_{d,P\;501}-i_{d,N\;501}$$

where *i_d,P501_* and *i_d,N501_* are currents flowing through MP501 and MN501, respectively. Finally, we can derive the OTA's transconductance as follows.

(6) $$g_{m,\text{OTA}}=\frac{i_{o}}{\text{VP}-\text{VN}}$$

However, the transconductance of Gm1 can be adjusted by tuning the bias, DAC_out. Referring to Figure 12, Gm1-C1 and Gm2-C2 constitute a 2nd-order RC oscillator with a positive feedback to generate an oscillation signal. On the other hand, Gm3 and Gm4 are used to keep the peak-to-peak amplitude of the generated sine wave. The frequency tuning range of tunable OTA-C oscillator (shown in Figure 13) is limited from 2 MHz to 10 MHz according to the characteristics of the FPW allergy biosensor.

#### Peak Detector

The output signal of each FPW allergy biosensor in Figure 8 will reach its peak voltage when the input frequency is equal to the resonant frequency. A peak detector is, then, used to detect the maximum peak from the output of FPW allergy biosensor and to further determine the corresponding frequency.

**Figure 14.**
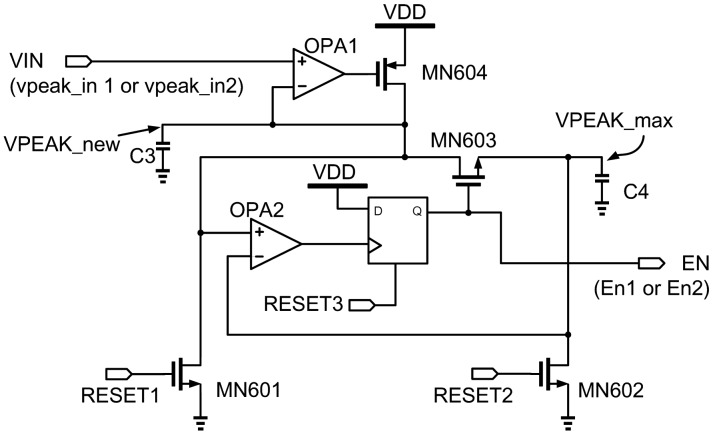
Schematic of the peak detector.

Figure 14 shows the proposed peak detector and the detailed operating steps are listed as follows:

*Step 1*: Initially, RESET1, RESET2, and RESET3 are biased at high to discharge C3, C4 and reset the D flip-flop.
*Step 2*: The sine wave from FPW allergy biosensor's output is fed to VIN (vpeak_in1 or vpeak_in2 in Figure 8). When VIN is higher than VPEAK_new, OPA1 will turn on MN604. Then, C3 is charged until VPEAK_new = VIN.
*Step 3*: MN603 is off to isolate VPEAK_new from VPEAK_max. If VPEAK_new is higher than VPEAK_max, OPA2 will trigger the D flip-flop. Then, EN (En1 or En2 in Figure 8) is pulled high to turn MN603 on. Hence, VPEAK_max is pulled close to VPEAK_new through MN603. If VPEAK_new is not higher than VPEAK_max, VPEAK_$max keeps the prior high voltage value.
*Step 4*: When VPEAK_max is equal to VPEAK_new, RESET3 will be pulled up high to resetthe D flip-flop to set EN = 0. VPEAK_new and VPEAK_max are isolated again by MN603.

By the above steps, the peak detector can generate the enable signals, EN (En1 or En2 in Figure 8), to enable the registers (reg1 or reg2 in Figure 8), respectively, and store the respective counting numbers therein. By the subtraction the contents in reg1 from that in reg2, the frequency-shift variation, Δf, can be derived.

## 4. Implementation and Measurement

**Figure 15.**
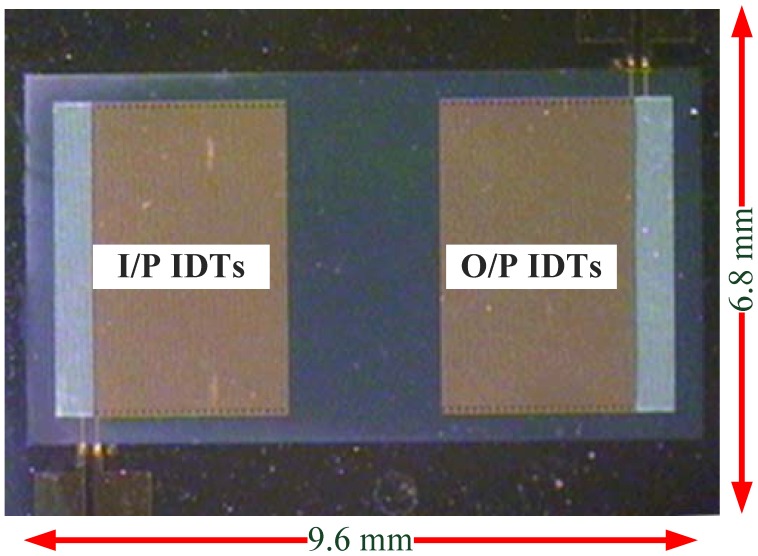
Photograph of the implemented FPW sensor.

Figure 15 shows the implemented FPW sensor using MEMS technology and the total fabrication processes, including six photolithography steps, eight thin-film deposition steps, and six etching/lift-off steps.

**Figure 16.**
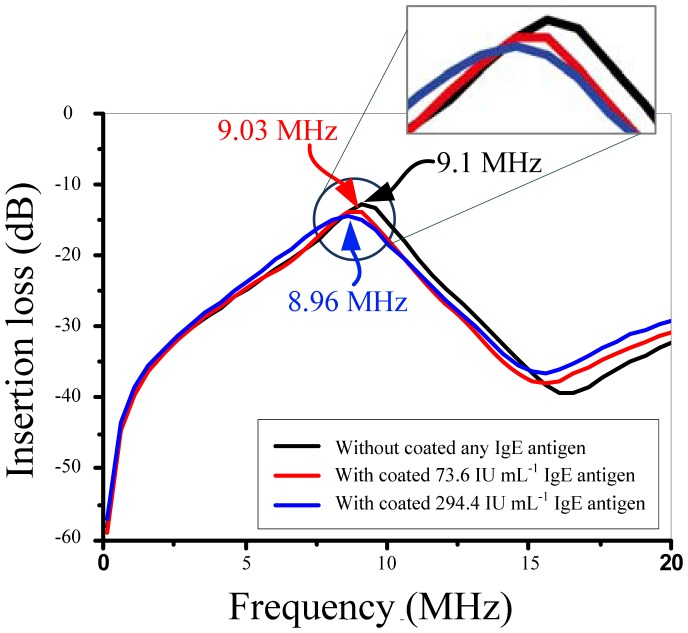
Frequency responses of the FPW allergy biosensor.

As the IgE antigen concentration of allergy patient is usually higher than 100 IU·mL^−1^ the FPW allergy biosensor developed in this study is coated with 73.6 IU·mL^−1^ (non-allergy) and 294.4 IU·mL^−1^ (allergy) IgE antigen and their frequency responses are measured. The center frequency of the implemented FPW allergy biosensor is measured by the Casecade RHM-06/V probe station and the HP8714ET network analyzer. Two cascade coplanar 150-GSG probes are used to be coupled with the input IDTs and output IDTs of the FPW sensor, respectively. Notably, all the measurements are proceeded at the room temperature. As shown in Figure 16, the center frequency of the implemented FPW device is only about 9.1 MHz, which is very easy for developing a readout circuit using a CMOS process. Compared with the previous research [26], this FPW device has adopted a pair of RGE microstructures to dramatically reduce the insertion loss from −50 dB to −13 dB. The low center frequency and low insertion loss advantages are beneficial to the development of the signal processing circuit for FPW-IgE microsensors. In an early preliminary experimental measurement, 70 kHz and 140 kHz frequency shifts are detected, respectively, as the 73.6 IU·mL^−1^ and 294.4 IU·mL^−1^ IgE antigen are coated on the FPW allergy biosensor. These results were consistent with the acoustic wave sensing theory that predicts a higher detecting sample mass will result in a higher frequency shift [27].

**Figure 17.**
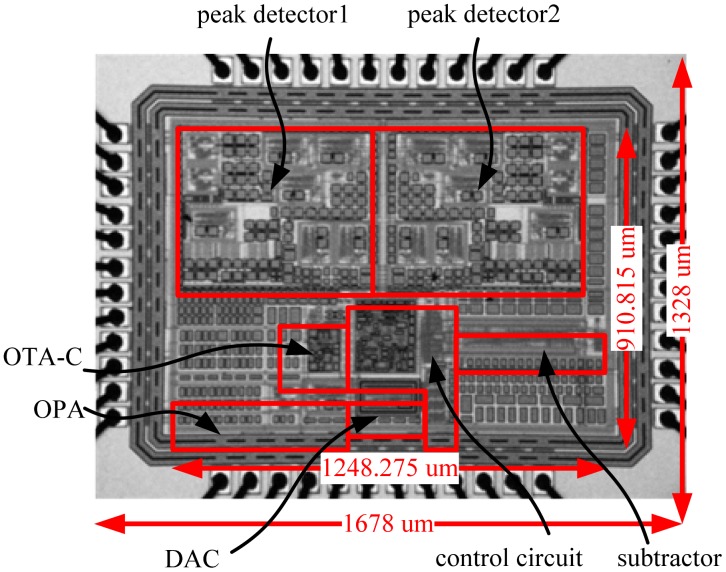
Die photo of the proposed frequency-shift readout circuit.

The proposed frequency-shift readout circuit for FPW allergy biosensors is fabricated on silicon using Taiwan Semiconductor Manufacturing Company (TSMC) standard 0.18 μm CMOS technology. Figure 17 shows the die photo including I/O PADs of the proposed prototype chip. The chip area of the proposed frequency-shift readout circuit is 1,678 × 1,328 μm^2^. We also carry out the detailed measurements for each subcircuit in the frequency-shift readout circuit to show the performance.

**Figure 18.**
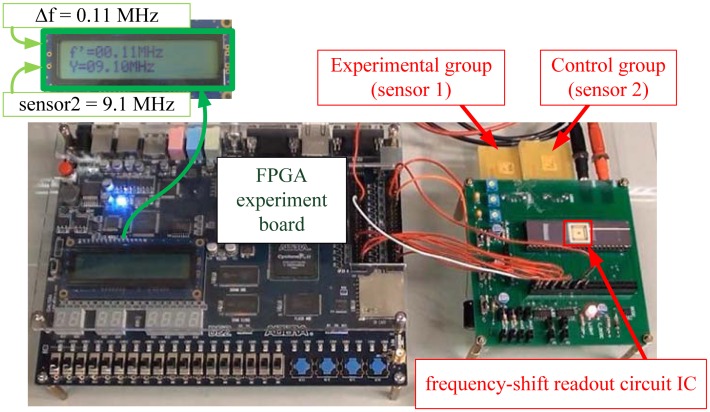
Prototype of the protein concentration measurement system.

The FPW sensors are coupled to the proposed readout system as shown in Figure 18. We utilize an FPGA (field-programmable gate array) experiment board to generate the control signals for the proposed frequency-shift circuit. When the frequency-shift measurement is done, the amount of the frequency-shift will be displayed on the LCD monitor. For instance, the LCD monitor displays the resonance frequency of the Control group FPW allergy biosensor (sensor2) is 9.1 MHz and the amount of the frequency shift of the Experimental group FPW allergy biosensor (sensor1) is 0.11 MHz. Thus, we can derive the concentration of IgE protein by a frequency to concentration conversion table.

By measurement, the frequency resolution of the proposed system must be smaller than 35 kHz. The typical 8-bit counter can generate a 0 to 256 counting signal to drive the DAC. Based on the output digital code of the counter, a corresponding bias voltage generated by the DAC is given to the OTA-C oscillator, which is in charge of delivering a 2 MHz to 10 MHz sine wave. By calculation, the frequency step of the proposed linear frequency generator is 31.25 kHz, which provides enough frequency resolution for the proposed system. When the input frequency equals to the resonant frequency, the output signal of the FPW allergy biosensor reaches the peak voltage. The accuracy of the proposed peak detector is measured to be 15 mV.

**Figure 19.**
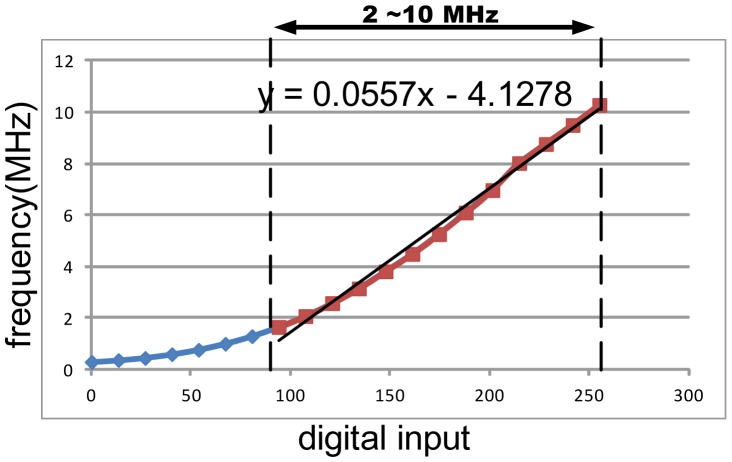
Curve of frequency *versus* counter's count generated by OTA-C oscillator.

**Figure 20.**
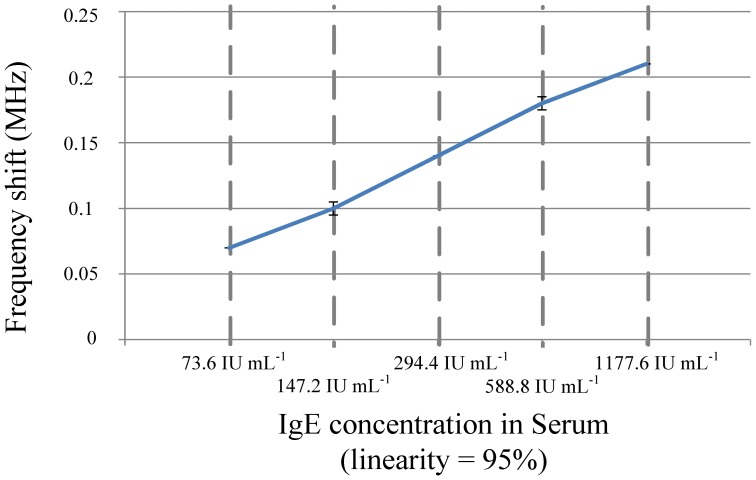
Frequency shift *versus* IgE concentration.

Since the INL and DNL are measured to be both smaller than 0.5 least significant bits (LSB), the proposed DAC with the current complementary circuit is able to provide an accurate voltage output to the OTA-C oscillator. The measurement results of the OTA-C oscillator is illustrated in Figure 19, which shows the frequency output almost linearly proportional to the output of the counter, particularly in the range from 50 to 250. The curve of the frequency shift *versus* the IgE concentration in human serum is shown in Figure 20. Notably, the linearity is better than 95%. Observably, the curve is approximate linearity.

**Figure 21.**
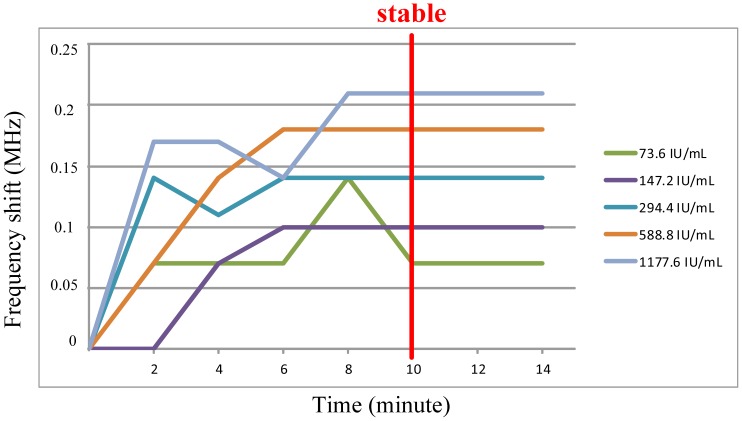
Frequency shift of the FPW allergy biosensor coated different IgE concentrations in human serums.

Figure 21 shows the timing behavior of the FPW allergy biosensor coated with different IgE concentrations in human sera. As shown in this figure, the frequency shift of each IgE concentration is stable after 10 min, which is faster than any existing IgE measurement systems, including ELISA, CLIA, and Abbott AxSYM, *etc.*

**Figure 22.**
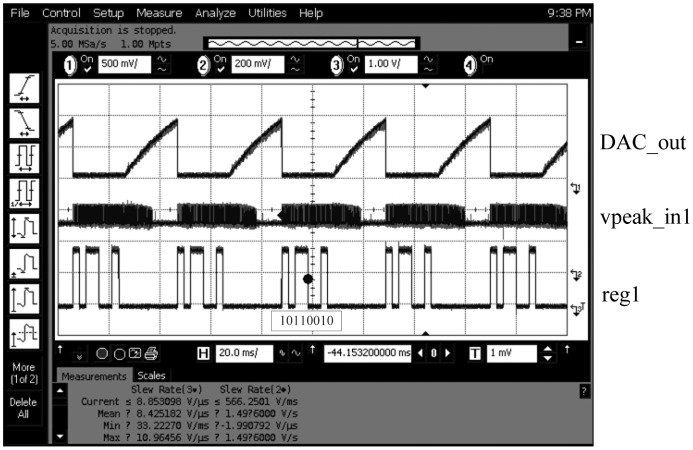
Measurements of the frequency-shift readout circuit.

Figure 22 illustrates the timing measurements of DAC_out, vpeak_in1, and the value of reg1, respectively. When the 8-bit counter begins counting, the output of the DAC, DAC_out, will generate a linearly rising signal sent to the OTA-C oscillator. The OTA-C oscillator is, then, used to generate a sine wave signal with the frequency from 2 MHz to 10 MHz to sensor1 (and sensor2 as well at the same time). When the input frequency equals to the resonant frequency of the sensor1, vpeak_in1 will reach a peak value. The reg1 stores the counting number given by the 8-bit counter corresponding to the resonant frequency of the sensor1. The power consumption of the proposed frequency-shift readout circuit is measured to be 48 mW at a 0.1 MHz clock. The comparison with a similar prior work is tabulated in Table 3.

## 5. Conclusions

This paper presents a frequency-shift readout circuit and system for a two-port FPW allergy biosensor. The linear frequency generator generates a linear frequency sweep fed into the FPW allergy biosensor. The peak detectors are used to detect the resonant frequencies of the Experimental group and Control group of the two-port FPW allergy biosensors. The detected resonant frequencies are stored in the registers, reg1 and reg2, respectively. The frequency-shift amount is measured by the proposed prototype system correctly. The proposed prototype system just needs less than 10 min operation time to produce the protein concentration measurement result. Furthermore, the proposed prototype system is very cost effective compared to the traditional methods and existing systems. Therefore, the proposed technique should be an attractive solution for protein concentration measurement.

## Figures and Tables

**Table 1.** Comparison with commercial allergy detectors and the proposed FPW-IgE microsystem.

	**ELSA**	**CLIA**	**Abbot AxSYM (FPIA)**	**FPW-based Microsystem**
Method	Enzyme Immunoassay Colorimetric	Enzyme Immunoassay, Chemiluminescence	Automated Latex Enhanced Immunoassay	Acoustic/piezoelectric Microsensor
Principle	Streptavidin Biotin Based Sandwich Assay	Streptavidin Biotin Based Sandwich Assay	Polystyrene Latex Agglutination	Flexural Plate-wave Based Mass Sensing
Calibrators (IU·mL^−1^) (in human serum)	0, 5, 25, 50, 150 (IRP 75/502)	0, 5, 25, 50, 150, 400 (IRP 75/502)	0, 50, 100, 200, 500, 1,000	0, 73.6, 147.2, 294.4, 588.8, 1,177.6
Sample (μL/well)	25	25	1,500	<5
Total operation time (min)	120–150	120–150	60–120	<10
Sensitivity (IU·mL^−1^)	1.0	1.0	1.0	1.0
Safety	No radioactive or toxic waste	No radioactive or toxic waste	No radioactive or toxic waste	No radioactive or toxic waste
Equipment cost (USD)	∼10,000	∼23,000	∼100,000	∼150
Dimension (cm^3^)	∼40 × 30 × 11	∼40 × 30 × 33	∼160 × 85 × 152	<25 × 10 × 5
Disposable	Yes	Yes	Yes	Yes

**Table 2.** Specifications of the FPW-based allergy biosensor.

**Chip size**	**9.6 mm × 6.8 mm**
Wavelength (Periodicity)	Enzyme Immunoassay Colorimetric
No. of finger pairs	25
Finger width/finger spacing	20 μm/20 μm
Acoustic aperture	3.2 mm
Acoustic path length	3.58 mm
Center frequency	9.1 MHz

**Table 3.** Comparison with prior work.

	**Proposed**	**[31]**
Implementation technique	system on chip	PCB discretes
Measurement method	peak detection	phase detection
Process (μm)	0.18	N/A
Supply voltage (V)	1.8	+5, −5, and 3.3
Frequency (MHz)	0.1	4.2
Power (mW)	48	N/A
Year	2012	2008
